# An intraocular oxygenated emulsion suppresses retinal fibrosis by inhibiting hypoxia-driven bioenergetic shifts and mesenchymal transformation

**DOI:** 10.21203/rs.3.rs-9888803/v1

**Published:** 2026-06-12

**Authors:** Binapani Mahaling, Lei Xi, Qiaoran Qi, Maryam Shayan, Anil Upreti, Zhirong Lin, Asmaa Zidan, Qiurong Zhu, Olivia Qin, Kathryn Pate, Leo Kim, Jia Yin, Yihe Chen

**Affiliations:** Schepens Eye Research Institute of Mass Eye and Ear and Department of Ophthalmology, Harvard Medical School; Schepens Eye Research Institute of Mass Eye and Ear and Department of Ophthalmology, Harvard Medical School; Schepens Eye Research Institute of Mass Eye and Ear and Department of Ophthalmology, Harvard Medical School; Schepens Eye Research Institute of Mass Eye and Ear and Department of Ophthalmology, Harvard Medical School; Schepens Eye Research Institute of Mass Eye and Ear and Department of Ophthalmology, Harvard Medical School; Schepens Eye Research Institute of Mass Eye and Ear and Department of Ophthalmology, Harvard Medical School; Schepens Eye Research Institute of Mass Eye and Ear and Department of Ophthalmology, Harvard Medical School; Schepens Eye Research Institute of Mass Eye and Ear and Department of Ophthalmology, Harvard Medical School; Schepens Eye Research Institute of Mass Eye and Ear and Department of Ophthalmology, Harvard Medical School; Coruna Medical, LLC; Schepens Eye Research Institute of Mass Eye and Ear and Department of Ophthalmology, Harvard Medical School; Schepens Eye Research Institute of Massachusetts Eye and Ear; Schepens Eye Research Institute of Mass Eye and Ear and Department of Ophthalmology, Harvard Medical School

## Abstract

Fibrosis drives progressive organ dysfunction, yet targeted therapies remain limited. In the eye, proliferative vitreoretinopathy (PVR) is a blinding fibrotic disease without approved medical treatment. Here, single-cell RNA sequencing of human PVR membranes revealed convergent activation of hypoxia-responsive programs across major cell populations. Using a molecular probe, we directly validated spatial and temporal hypoxia in an open-globe injury model that recapitulates traumatic PVR. Intravitreal delivery of a supersaturated oxygen emulsion (SSOE) preserved retinal function while reducing fibrocellular membrane formation and inflammation. Mechanistically, human PVR cells exhibited metabolic shifts toward glycolysis alongside epithelial-mesenchymal transition (EMT). SSOE corrected cellular hypoxia, preserved mitochondrial integrity, suppressed glycolytic shift, and inhibited EMT in human retinal pigment epithelial cells, while reducing spontaneous contractility in primary human PVR cell cultures. Together, these findings identify hypoxia as a critical driver of retinal fibrosis and support localized intraocular oxygenation as a viable therapeutic strategy for preventing fibrotic vision loss.

## INTRODUCTION

Retinal fibrosis is a major cause of vision loss in blinding disorders, including proliferative vitreoretinopathy (PVR), diabetic retinopathy (DR), age-related macular degeneration (AMD), and ocular trauma. Aberrant fibrocellular scarring disrupts retinal architecture and neuronal function, yet no targeted therapy currently exists to prevent or reverse retinal fibrosis^1–3^. Because ocular fibrosis stems from dysregulated tissue repair characterized by persistent inflammation and excessive proliferation, current clinical management relies on non-specific anti-inflammatory or antiproliferative agents. However, corticosteroids provide inconsistent antifibrotic benefit^4–6^ and are limited by severe ocular adverse effects^7,8^, whereas antiproliferative agents such as mitomycin C, 5-fluorouracil, and methotrexate exhibit variable efficacy and substantial toxicity^1,9^. Consequently, surgical removal of scar tissue remains the standard intervention for advanced disease, but fails to address the underlying pathogenic mechanisms and is frequently followed by incomplete visual function recovery^2^ and recurrent fibrosis^10,11^.

Efforts to develop mechanism-based therapies have focused on intercepting key molecular mediators of intraocular fibrosis. While elevated intraocular levels of vascular endothelial growth factor (VEGF) have been reported in fibrotic eye diseases, anti-VEGF therapies have shown mixed efficacy in glaucoma surgery- and AMD-related fibrosis and no benefit in PVR^1,9,12^. Similarly, transforming growth factor-β (TGF-β), a potent inducer of myofibroblast differentiation, and its signaling cascade have been extensively targeted preclinically; however, clinical blockade of TGF-β failed to improve fibrotic outcomes following glaucoma surgery^9^, despite a recent preliminary conference report suggesting potential benefit in fibrosis associated with DR and AMD^13^. Together, these limited therapeutic successes suggest that VEGF and TGF-β may function primarily as downstream effectors within complex fibrotic networks, highlighting the need to identify upstream disease drivers that coordinate broader, multifaceted profibrotic programs.

Inflammation and ischemia are hallmarks of fibrotic eye diseases^14^. Infiltrating immune cells and reparative tissues drastically increase metabolic demand, while vascular disruption limits oxygen delivery, together generating a hypoxic microenvironment. Emerging evidence implicates hypoxia and hypoxia-inducible signaling in ocular fibrosis^11,14–18^ and in broader fibrotic remodeling across multiple organs^19,20^, raising the possibility that cellular hypoxia functions as an upstream regulator coordinating profibrotic transcriptional and metabolic networks. Although systemic oxygen supplementation can promote organized wound healing in other tissues^21,22^, systemic delivery carries significant risks of off-target hyperoxia, and whether correcting local intraocular oxygen deficits can therapeutically suppress retinal fibrosis remains unknown.

Here, we investigated the pathogenic role of cellular hypoxia in PVR, a severe form of retinal fibrosis, using human single-cell transcriptomics, a clinically relevant open-globe injury (OGI) model, human retinal pigment epithelial (RPE) cell and primary PVR cell cultures, and localized intraocular oxygen supplementation. PVR develops following retinal injury triggered by trauma, non-traumatic conditions, or surgical intervention. Recent work has implicated hypoxia-associated transcriptional programs in PVR^23^, supporting a potential role for oxygen dysregulation in disease progression. However, the precise spatial and temporal dynamics of tissue hypoxia during PVR development, its direct pathogenic relevance and mechanisms in clinically representative settings, and its therapeutic actionable potential have remained incompletely defined.

To bridge these gaps, we first confirmed hypoxia-responsive programs in an independent human PVR single-cell RNA sequencing (scRNA-seq) dataset and then directly visualized early tissue hypoxia *in vivo* using a molecular hypoxia probe in the OGI model that faithfully recapitulates traumatic PVR. To counteract this local oxygen deficit, we introduced a localized oxygen delivery strategy using an intravitreal supersaturated oxygen emulsion (SSOE), adapted from a prior ocular surface formulation^24^, and evaluated its effects through longitudinal structural and electroretinographic functional analyses. Mechanistically, global pathway analysis identified epithelial-mesenchymal transformation (EMT) and glycolytic reprogramming as dominant features of human PVR membrane cells. We further demonstrated that SSOE corrected cellular hypoxia, preserved mitochondrial integrity, inhibited the glycolytic shift and EMT in human RPE cells, while reducing the pathogenic contractility of primary human PVR cell cultures. Together, our findings establish cellular hypoxia as a critical, early, upstream driver of retinal fibrosis and support localized tissue oxygenation as a viable therapeutic strategy to prevent fibrotic vision loss.

## RESULTS

### Hypoxia emerges early and persists across PVR cell states

**Figure 1:**
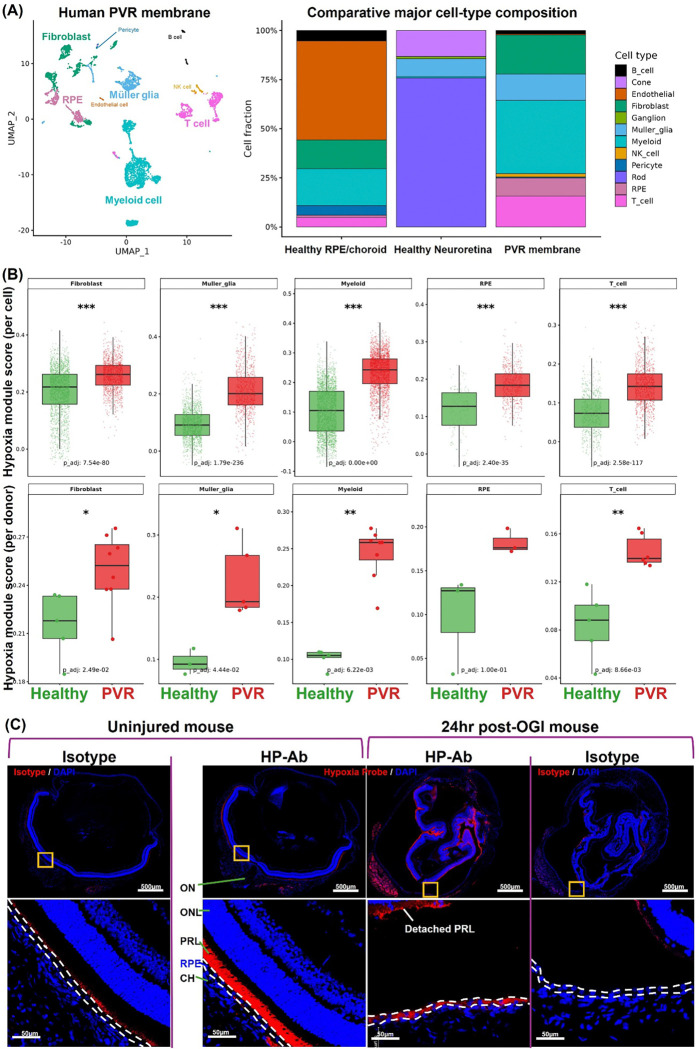
Cellular hypoxia is involved in both established human PVR membranes and early after injury that induces PVR in mice. **(A)** Global cell-type composition and major cell-class structure in the PVR membrane scRNA-seq dataset. The UMAP projection displays the cell-type composition in human PVR membranes. The stacked bar plot displays the fractional composition (proportions summing to 100%) of the unified major cell types across PVR membrane, healthy neuroretina, and healthy RPE/choroid datasets, allowing for comparison independent of total cell number. **(B)** The hallmark hypoxia program is elevated across major cell classes in human PVR membranes compared with healthy references. Boxplots show per-cell (upper panel) and per-donor (lower panel) hypoxia module scores (y-axis) computed from the MSigDB Hallmark Hypoxia gene set and summarized within each major cell class. Cells from healthy datasets (Müller glia from healthy neuroretina dataset, and the rest from healthy RPE/choroid dataset) are shown in green and PVR cells in red. Each dot represents an individual cell (per-cell analysis) or an individual donor (per-donor analysis); box center line indicates the median, boxes the interquartile range (IQR), and whiskers extend to 1.5×IQR. Donor medians were retained only when donors contributed at least 10 cells in that category. Statistical significance for PVR vs Healthy within each cell class is indicated above each panel, and multiple-testing-adjusted p-values (p_adj) are shown within panels. **(C)** A mouse model of open-globe injury (OGI)-induced PVR exhibits rapid and extensive induction of cellular hypoxia in RPE layer. Representative images show pimonidazole staining (HypoxyProbe, HP) in eyeball cross-sections from normal uninjured and 24 hours post-injury mice, respectively. HP detects O_2_ partial pressures of < ~1% O_2_. Note the stained photoreceptor outer segment in uninjured eye separated from the RPE layer in the OGI eye. Enlarged focal images show significantly increased HP staining within RPE layer (bounded by two white dashed lines with a linear arrangement of single-layer nuclei) in injured eye compared to uninjured control. ON, optic nerve; ONL, outer nuclear layer; PRL, photoreceptor layer; RPE, retinal pigment epithelium; CH, choroid. The images shown are representative of one of 3 experiments performed.

The retina comprises the neuroretina, which processes visual signals, and the underlying RPE layer, which provides essential metabolic and trophic support to the neuroretina. The RPE overlies the choroid, a highly vascularized plexus rich in circulating immune cells that supplies oxygen to both the RPE and the outer retina^25^. Disruption of this architecture is a defining feature of PVR. To determine whether cellular hypoxia characterizes human PVR, we performed integrated dimensionality reduction and Uniform Manifold Approximation and Projection (UMAP) analysis on a published scRNA-seq dataset of surgically excised human PVR membranes from patients with grade C disease (Broad Institute: SCP2582)^26^. These data were integrated with reference datasets from healthy human RPE/choroid tissue (GEO: GSE135922)^25^ and healthy neuroretina (ArrayExpress: E-MTAB-7316)^27^. Across the three datasets, cell-type composition aligned with expected tissue biology, and UMAP clustering demonstrated clear separation of major cell lineages, validating our annotation framework. Healthy RPE/choroid was enriched for vascular, stromal, and immune populations, predominantly endothelial cells, myeloid cells, fibroblasts, and pericytes, with smaller fractions of RPE and lymphocyte populations (Supplementary Fig. 1 and Fig. 1A). Healthy neuroretina was dominated by neuronal populations, with rod photoreceptors constituting the largest fraction, and smaller contributions from other expected retinal cell classes (e.g., cone-related and inner-retina neuronal classes, retinal progenitor-like cells, and Müller glia) (Supplementary Fig. 1 and Fig. 1A). In contrast, PVR membrane exhibited a fibrotic and immune-skewed cellular ecosystem, characterized by a massive expansion of fibroblast/stromal and myeloid populations, alongside lymphocytes, epithelial cells, and activated Müller glia (Fig. 1A). Hypoxia-associated transcriptional programs were broadly elevated across all major PVR cell populations relative to lineage-matched healthy controls. The most pronounced upward shifts occurred in the myeloid compartment, with robust increases also detected in RPE, Müller glia, fibroblasts, and T cells (Fig. 1B). These findings demonstrate that hypoxia represents a shared feature of the principal disease-driving cell types within established human PVR membranes.

To directly determine whether cellular hypoxia arises early during PVR development, we examined a highly clinically relevant mouse model of OGI (Supplementary Fig. 2), in which tractional fibrocellular PVR membranes reliably form by 3 weeks post-injury. This model more faithfully recapitulates the developmental process of human traumatic PVR without artificial introduction of exogenous cells or chemicals into the vitreous cavity required by other models^28^. A molecular hypoxia probe (pimonidazole) administered 24 hours post-injury revealed acute retinal detachment (separation of the neuroretina from the underlying RPE) accompanied by localized induction of tissue and cellular hypoxia. Hypoxic signals were most prominent within the detached RPE layer and the peripheral retina adjacent to the injury site, spatially coinciding with early immune cell infiltration (Fig. 1C), a critical initial event in PVR development^2,11,29^. Together, these findings establish cellular hypoxia as an early and sustained feature of PVR pathogenesis.

### SSOE is biocompatible and safe for intraocular application

**Figure 2:**
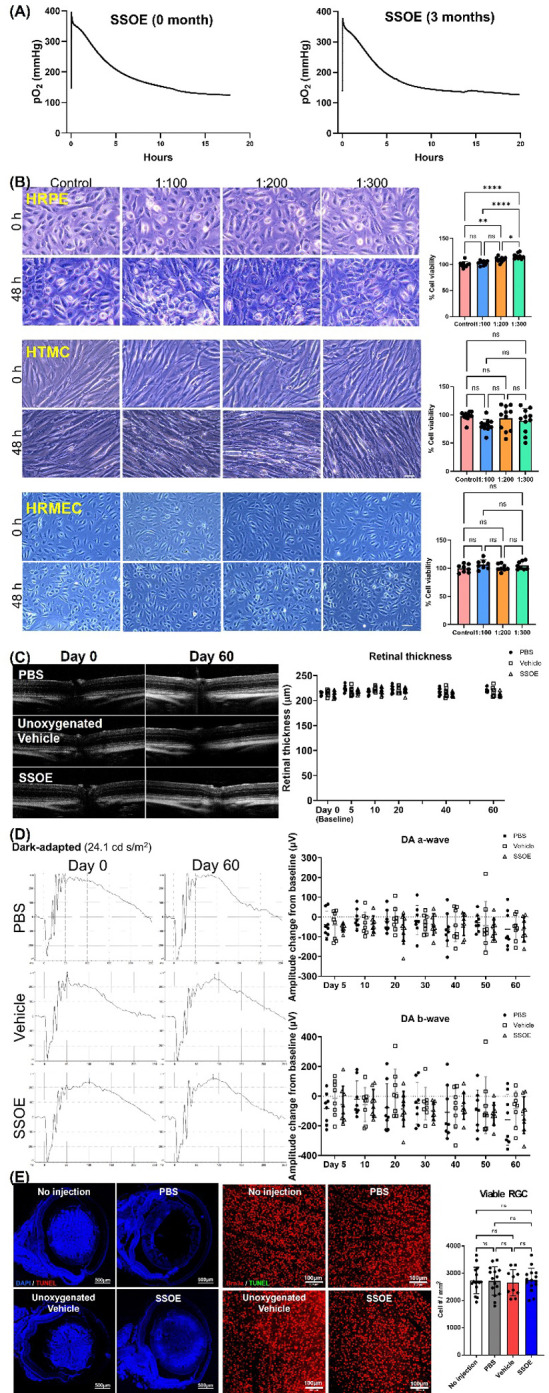


The SSOE technology is a perfluorodecalin (PFD)-based formulation designed for localized oxygen delivery^24^. PFD is an established oxygen carrier and has been used clinically as a vitreoretinal surgical adjuvant^30^. While ophthalmic formulations containing 25% (w/v) PFD promote organized ocular surface wound healing without toxicity^24,31^, intraocular delivery following penetrating trauma poses introduces unique safety considerations. These include potential mechanical retinal damage from pure PFD (100%) due to gravity-dependent density differences^32^ and the distinct oxygen sensitivities of retinal versus corneal tissues^33,34^. To optimize intraocular safety, the SSOE was reformulated using 15% PFD in the current study. Upon ambient air exposure, this adjusted formulation exhibited an initial oxygen release peaking at a partial pressure (pO_2_) of approximately 400 mmHg, followed by sustained release over ~15 hours before equilibrating to atmospheric levels (~160 mmHg). This peak pO_2_ was lower than that of the original 25% PFD formulation (>600 mmHg)^24^, minimizing the risk of hyperoxic toxicity to the sensitive retina. Unopened SSOE retained a comparable oxygen release profile after 3 months of storage, confirming formulation stability (Fig. 2A).

We next evaluated SSOE biocompatibility *in vitro* using primary human intraocular cell types essential to ocular homeostasis: RPE cells, trabecular meshwork cells, and retinal microvascular endothelial cells. Across a broad concentration range, SSOE did not significantly alter cell morphology or reduce viability in any cell type (Fig. 2B and Supplementary Fig. 3). Interestingly, while the unoxygenated vehicle control reduced RPE and trabecular meshwork cell viability at the highest tested concentration (1:100, v/v), it had no effect on retinal microvascular endothelial cells (Supplementary Fig. 4). These findings suggest that oxygenation mitigates vehicle-associated cytotoxicity. To eliminate any confounding vehicle effects, a conservative 1:200 dilution was selected for subsequent *in vitro* mechanistic experiments.

To assess *in vivo* safety, healthy mouse eyes received a single 1 μL intravitreal injection of SSOE, unoxygenated vehicle, or phosphate-buffered saline (PBS). Retinal structure and function were monitored longitudinally for 60 days using optical coherence tomography (OCT) and electroretinography (ERG). OCT imaging revealed no abnormalities in vitreous or retinal architecture across all groups, and retinal thickness did not differ at any time point (Fig. 2C). ERG analysis showed no significant differences in changes from baseline for dark-adapted (scotopic) a-wave or b-wave amplitudes, or light-adapted (photopic) b-wave amplitudes among the groups (Fig. 2D and Supplementary Fig. 5A). Furthermore, the implicit times for all ERG components remained unchanged, confirming that SSOE does not delay the kinetic speed of the retinal electrical response in normal eyes (Supplementary Fig. 5B). Histological evaluation at day 60 confirmed intact ocular structures without inflammatory infiltrates (Supplementary Fig. 5C), and TUNEL staining showed no increase in apoptotic cells within retinal nuclear or ganglion cell layers (Fig. 2E). These results demonstrate that SSOE is well tolerated by ocular tissues over extended periods and does not impair retinal structure or function *in vivo*.

### Local SSOE treatment preserves retinal function and suppresses retinal fibrosis in a traumatic PVR model

**Figure 3:**
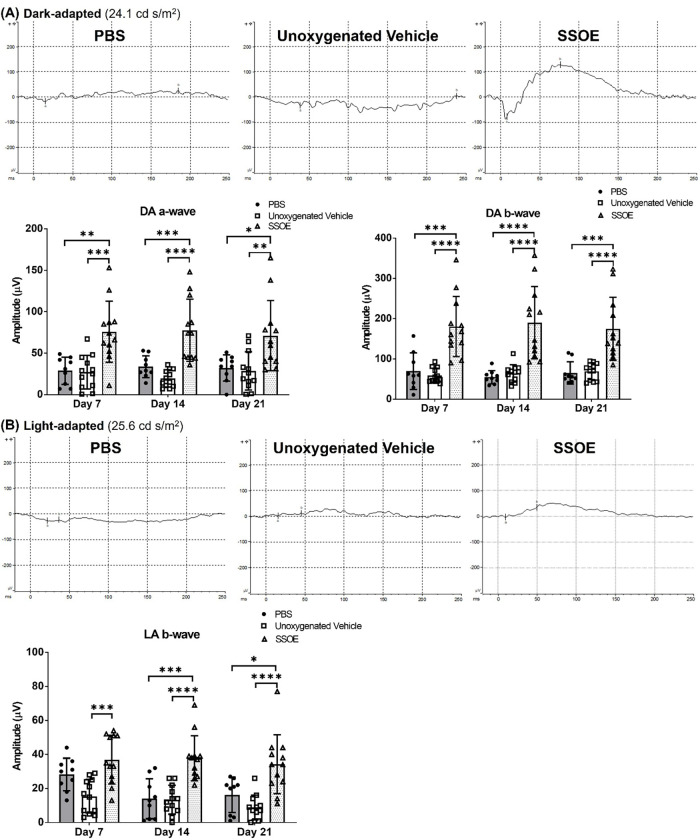
Local SSOE treatment effectively preserves retinal function after OGI in mice. Mice with OGI were locally treated with a single dose of PBS, unoxygenated vehicle, or SSOE after injury, and followed for 21 days. Retinal function was evaluated by full-field ERG *in vivo*. Representative recordings to light stimuli at 24.1 and 25.6 cd s/m^2^ for scotopic dark- (DA) **(A)** and photopic light-adapted (LA) **(B)** eyes, respectively, are shown on the upper panels, and amplitudes of a-wave and b-wave are depicted in bar charts on the lower panels. N = 9–12/group pooled from four independent experiments. *P < 0.05, **P < 0.01, ***P < 0.001, and ****P < 0.0001 by one-way ANOVA with Tukey’s post hoc multiple comparisons test.

We next evaluated the therapeutic efficacy of localized SSOE administration in our mouse model of traumatic PVR. Immediately following OGI, eyes received an intravitreal injection of SSOE, unoxygenated vehicle, or PBS, and retinal function was assessed longitudinally by ERG for 21 days. By day 7 post-injury, both control groups exhibited profound declines in ERG response amplitudes. Conversely, SSOE-treated eyes retained significantly higher amplitudes of both dark-adapted and light-adapted waves, a functional preservation that persisted throughout the 21-day study (Fig. 3). Furthermore, while control eyes displayed prolonged implicit times in dark-adapted responses and shortened implicit times in light-adapted responses, SSOE-treated eyes showed progressive kinetic normalization, returning to values comparable to uninjured eyes by day 21 (Supplementary Fig. 6).

**Figure 4:**
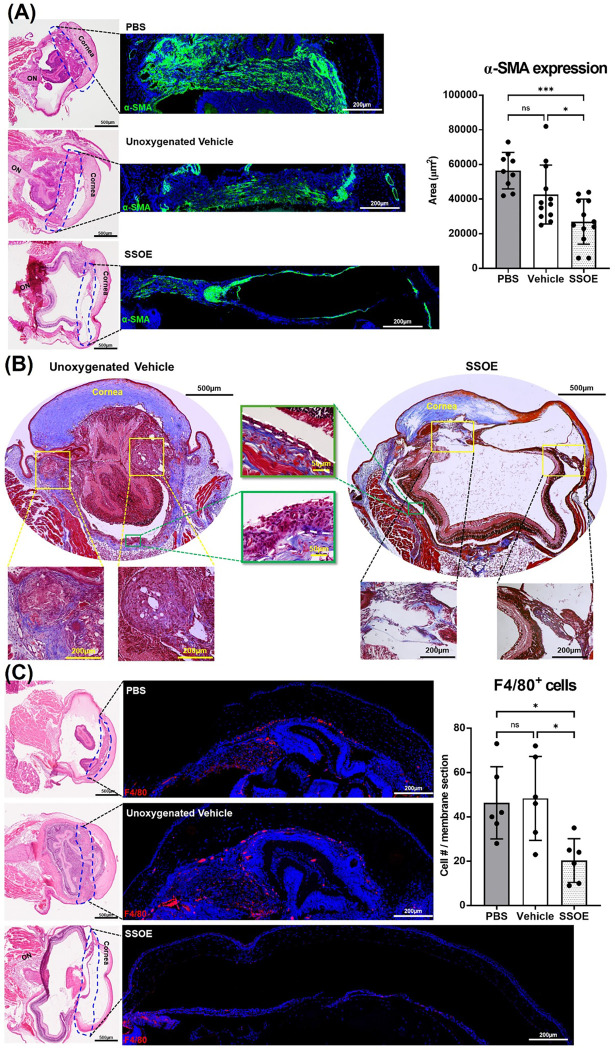
Local SSOE treatment effectively preserves ocular structures and mitigates intraocular fibrocellular membrane formation in the mouse model of OGI-induced PVR. Mice with OGI were locally treated with a single dose of PBS, vehicle, or SSOE immediately after injury. At day 21 post-OGI, animals were euthanized, and eyeballs were collected for histopathological examination. **(A)** Representative images of H&E staining and immunofluorescence staining of α-SMA with a summary graph of α-SMA^+^ areas per membrane section. Both PBS and vehicle-treated groups exhibit severe retinal detachment and diffuse retinal folds of full-thickness, along with extensive formation of α-SMA^+^ fibrocellular membrane inside the eye, while SSOE-treated group exhibits only mild retinal detachment and folding, well-preserved retinal anatomy, intraocular cavity space and globe size, along with minimal formation of PVR membrane. ON, optic nerve. N = 9–12/group pooled from four independent experiments. **(B)** Representative images of Masson’s trichrome staining showing additional collagen deposition (blue) in PVR membranes and multilayered spindle-shaped cells extending from the original monolayer RPE (bounded by two white dashed lines in the green box inserts) in the vehicle group, which are much milder in the SSOE group. Images shown are representative of 3–4/group. **(C)** Representative images of H&E staining and immunofluorescence staining of F4/80 with a summary graph of F4/80^+^ cells per membrane section. SSOE-treated group exhibits well-preserved retinal anatomy, intraocular cavity space, and eyeball size similarly. N = 6/group pooled from two independent experiments. ns, not significant, *P < 0.05, and ***P < 0.001 by one-way ANOVA with Tukey’s post hoc multiple comparisons test.

Histological analysis at day 21 revealed severe PVR pathology in PBS- and vehicle-treated eyes, characterized by diffuse retinal detachment, full-thickness retinal folds, and extensive intraocular fibrocellular membranes. These membranes originated from the peripheral retina, expressed high levels of α-smooth muscle actin (α-SMA, a marker for myofibroblast), and exhibited dense collagen deposition, closely mirroring human grade C PVR^35^. These fibrotic structures connected with multilayered spindle-shaped cells at the periphery extending from central, multilayered, more RPE-like cells (Fig. 4A, B), indicating epithelial-mesenchymal transformation (EMT) whereby the normally quiescent, polarized RPE monolayer loses cell-cell adhesion, proliferates, and transdifferentiates into matrix-producing myofibroblasts^36–39^.

In sharp contrast, SSOE-treated eyes exhibited markedly less severe retinal detachment, mild retinal folding, better preservation of global eye size, and significantly less fibrocellular membrane formation (Fig. 4A, B). Immunostaining demonstrated a significant reduction in the total areas of α-SMA, collagen-I, and fibronectin expression in the SSOE group compared to both controls (Fig. 4A and Supplementary Fig. 7). Moreover, SSOE treatment blunted the accumulation of F4/80^+^ macrophage within the remaining fibrotic tissue (Fig. 4C), the predominant blood-borne myeloid population driving PVR fibrogenesis^40–42^. Together, these data demonstrate that local intraocular oxygenation preserves neuroretinal function, potently suppresses retinal fibrosis, and dampens macrophage infiltration *in vivo*.

### SSOE suppresses EMT of human RPE cells by correcting cellular hypoxia

**Figure 5:**
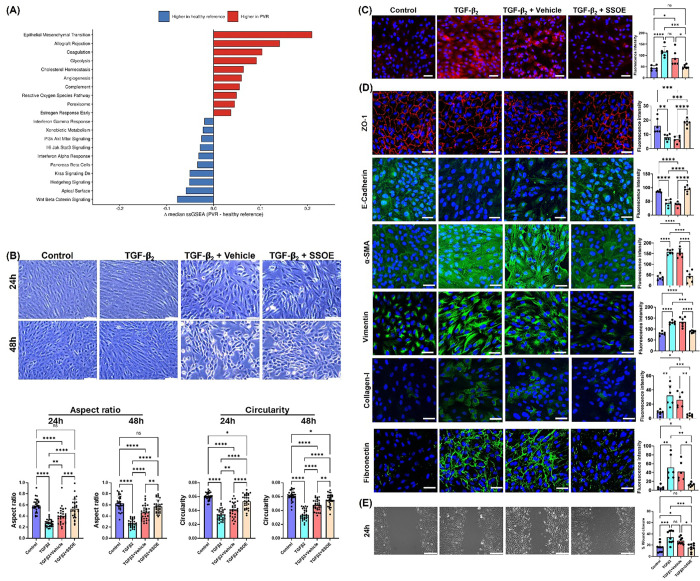
Human PVR cells show increased epithelial-mesenchymal transformation (EMT) activity and SSOE suppresses EMT of human APRE-19 cells *in vitro*. **(A)** Global donor-pseudobulk Hallmark pathway differences between human PVR and the matched healthy RPE/choroid reference. Donor-pseudobulk single-sample Gene Set Enrichment Analysis (ssGSEA) was performed across all Hallmark pathways using all cells from each dataset. Bars show the difference in median ssGSEA score between PVR and healthy reference, and the top positively and negatively shifted Hallmark pathways are displayed. Red bars indicate pathways higher in PVR, whereas blue bars indicate pathways higher in the healthy reference. **(B)** Morphological assessment of the cells at 24 and 48 hours using ImageJ analysis. Representative images are shown on the upper panel, with the cell outline selected using the polygon tool in ImageJ. Aspect ratio, calculated as the ratio of the minor to major axis, and cell circularity measurements are summarized in the lower panel. N = 30 cells from 3–4 biological replicates/group pooled from two independent experiments. Scale bars = 100 μm. **(C)** Immunofluorescence analysis of cellular hypoxic status using HypoxyProbe (HP) staining at 24 hours. Fluorescence intensity was measured from three images per sample, with 6 biological replicates/group pooled from two independent experiments. Scale bars = 50 μm.**(D)** Immunofluorescence analysis of epithelial markers, mesenchymal drivers, and extracellular matrix components at 48 hours. Fluorescence intensity was measured from three images per sample, with 6 biological replicates/group pooled from two independent experiments. Scale bars = 50 μm. **(E)** Cell migration was assessed using a scratch wound-healing assay. At cell confluence, a uniform scratch was introduced, and cells were cultured under the indicated conditions for 24 hours. Phase-contrast images were captured, and quantitative analysis of relative wound closure was performed by comparing wound areas at 24 hours with the baseline. Scale bars = 250 μm. ns, not significant, *P< 0.05, **P < 0.01, ***P < 0.001, and ****P < 0.0001 by one-way ANOVA with Tukey’s post hoc multiple comparisons test.

Our *in vivo* findings indicate that the therapeutic effects of SSOE stem primarily from the suppression of EMT in RPE cells. During EMT, RPE cells shed their epithelial characteristics, acquire migratory, contractile, and myofibroblast-like properties, and increase extracellular matrix (ECM) synthesis, generating tractional forces that drive membrane contraction and retinal detachment^43–45^. To map this unbiasedly, we performed comparative global hallmark pathway analysis on our integrated human scRNA-seq datasets. Among all modulated pathways, EMT represented the single largest upregulated program in human PVR membrane cells relative to healthy controls (Fig. 5A).

The inflammatory cytokine TGF-β is a central driver of EMT in RPE cells, and both TGF-β_1_ and TGF-β_2_-induced *in vitro* EMT models have been established in APRE-19 cells (a human RPE cell line)^46,47^. Consistently, PVR patients exhibited elevated vitreous levels of TGF-β, predominantly TGF-β_2_ and to a lesser degree TGF-β_1_, which correlate with disease severity^48^. Therefore, we employed a TGF-β_2_-induced *in vitro* EMT model to dissect the role of hypoxia. Addition of SSOE at the time of TGF-β_2_ induction effectively suppressed the morphological transition of RPE cells into elongated, spindle-shaped mesenchymal cells at both 24 and 48 hours. While the unoxygenated vehicle exerted a partial effect, it was consistently less efficacious than SSOE. Quantitative morphometric analysis showed that SSOE restored the cellular aspect ratio (width/length) to baseline control values and significantly improved cell circularity compared to vehicle treatment (Fig. 5B).

Importantly, TGF-β_2_ exposure rapidly induced a significant intracellular hypoxia in cultured RPE cells within 24 hours; this hypoxic state was completely reversed by SSOE, but not by the vehicle (Fig. 5C). Concurrently, TGF-β_2_ caused a profound loss of epithelial junctional proteins zona occludens-1 (ZO-1) and E-cadherin, with a reciprocal increase in the mesenchymal drivers α-SMA and vimentin, and the ECM components collagen-I and fibronectin. SSOE largely rescued epithelial marker expression and suppressed these mesenchymal and ECM programs (Fig. 5D). Furthermore, SSOE significantly blunted the accelerated cellular migration induced by TGF-β_2_ (Fig. 5E).

**Figure 6:**
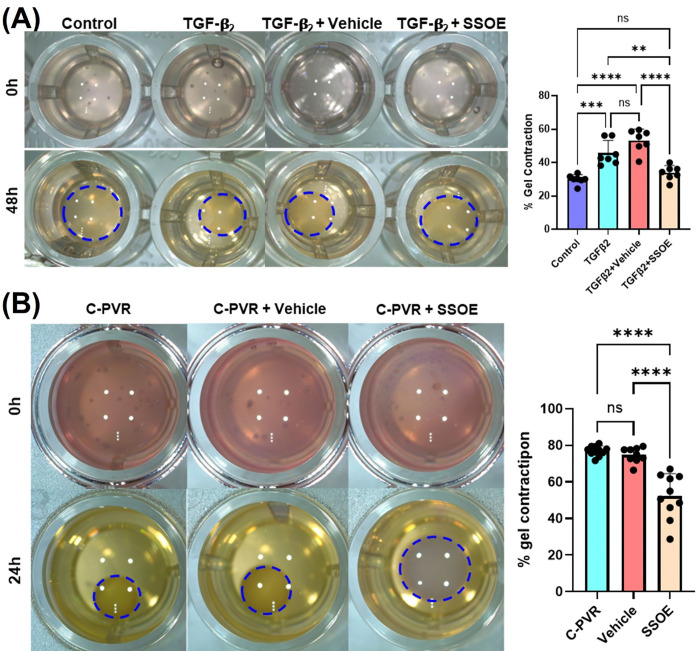
SSOE reduces cell contractility in both transformed human APRE-19 cells and primary human PVR cell cultures *in vitro*. The collagen-I gels containing APRE-19 cells **(A)** or primary human PVR cells **(B)** were subjected to the indicated treatments. Light microscopic images were captured at the indicated time points, with representative images showing contracted gels (within the blue dashed circle). The contraction percentage from the baseline (0 hour) is summarized in the bar charts. N = 7/group pooled from two independent experiments (A) and N = 9–11/group pooled from two independent experiments (B). ns, not significant, *P< 0.05, ***P < 0.001, and ****P < 0.0001 by one-way ANOVA with Tukey’s post hoc multiple comparisons test.

The acquisition of contractile force by transdifferentiated RPE cells is a critical myofibroblast trait that drives matrix remodeling, tissue stiffening, and tractional retinal detachment^49^. SSOE effectively mitigated TGF-β_2_-induced collagen gel contraction in ARPE-19 cells, whereas the vehicle had no significant impact (Fig. 6A and Supplementary Fig. 8). To validate this in a patient-derived context, we treated primary human PVR cells isolated from surgically excised fibrotic membranes. These primary cells exhibited rapid, robust spontaneous contraction of the collagen gel, much faster than the induced ARPE-19 line. SSOE treatment remarkably suppressed this contraction (Fig. 6B), demonstrating that local oxygenation not only prevents the initiation of RPE transdifferentiation but can also reverse the established profibrotic functional properties of fully formed ECM-producing cells.

### SSOE prevents mitochondrial disruption and glycolytic shift during EMT

Cells under hypoxic stress undergo metabolic adaptation to meet bioenergetic demands. EMT has been linked to mitochondrial disruption in tumor cells^50^, and EMT of RPE cells is associated with a shift away from oxygen-dependent mitochondrial oxidative phosphorylation (OXPHOS) toward oxygen-independent glycolysis^51^. Indeed, our single-cell transcriptomic analysis of human PVR membrane cells identified glycolysis as one of the top shifted metabolic pathways (Fig. 5A).

**Figure 7:**
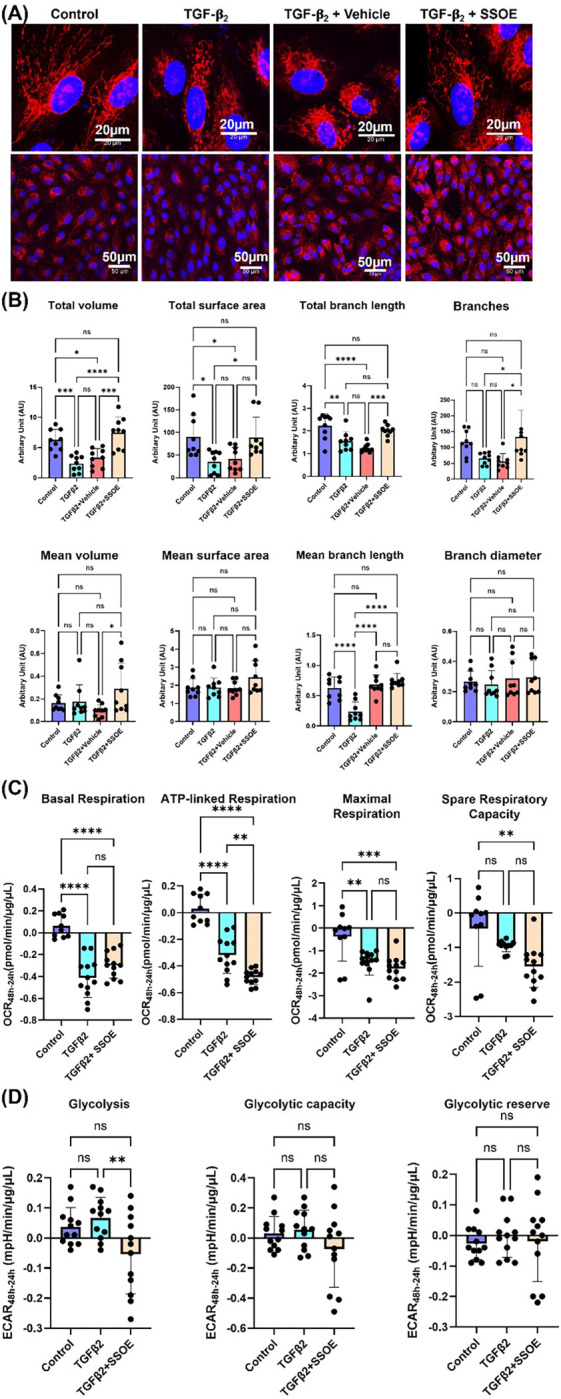
SSOE suppresses the disruption of mitochondrial structure and the shift in bioenergetic metabolism to glycolysis underlying human ARPE-19 transformation *in vitro*. **(A)** Representative images showing mitochondrial morphology using MitoTracker Orange CMTMRos (red) and DAPI (nuclei, blue) at 48 hours. **(B)** Unbiased three-dimensional quantification of mitochondrial shape and network features of MitoTracker Orange staining images. N = 9/group pooled from three independent experiments. **(C)** Changes of OXPHOS parameters, including basal respiration, ATP-linked respiration, maximal respiration, and spare respiratory capacity, at 48 hours from 24 hours via real-time measurement of oxygen consumption rate (OCR).**(D)** Changes of glycolytic function, including glycolysis, glycolytic capacity, and glycolytic reserve, at 48 hours from 24 hours via real-time measurement of extracellular acidification rate (ECAR). N = 10–12/group from one out of two independent experiments. ns, not significant, *P < 0.05, **P < 0.01, ***P < 0.001, and ****P < 0.0001 by one-way ANOVA with Tukey’s post hoc multiple comparisons test.

To visualize this under local oxygen modulation, we imaged the mitochondrial networks of human RPE cells *in vitro*. TGF-β_2_ exposure induced severe fragmentation of the mitochondrial network, causing organelles to lose their elongated structures and adopt a punctate, spherical morphology (Fig. 7A). SSOE treatment effectively preserved the networked, filamentous mitochondrial architecture, whereas the vehicle control did not (Fig. 7A). Unbiased three-dimensional quantification revealed that TGF-β_2_ significantly reduced total mitochondrial volume, total surface area, total branch length, and branch number, as well as mean branch length, without significantly altering individual organelle mean volume or branch diameter, indicating an overall loss of mitochondrial mass with relatively structural preservation of the remaining organelles. SSOE treatment effectively maintained all mitochondrial parameters at healthy control levels, while the vehicle rescued only the mean branch length (Fig. 7B).

We next functionally evaluated mitochondrial respiration and glycolytic activity using Seahorse analysis of oxygen consumption rate (OCR) and extracellular acidification rate (ECAR). At 24 hours post-induction, TGF-β_2_ significantly increased basal, ATP-linked, and maximal respiration, followed by a sharp exhaustion-like decline back to baseline levels by 48 hours (Supplementary Fig. 9A). Conversely, glycolytic parameters remained stable at 24 hours but rose by 48 hours following TGF-β_2_ treatment (Supplementary Fig. 9B), effectively recapitulating the chronic metabolic reprogramming seen in our human scRNA-seq analysis.

Evaluating the dynamic metabolic trajectory from 24 to 48 hours revealed that while control cells maintained steady-state OCR, TGF-β_2_-induced hypoxic cells showed steep declines in basal, ATP-linked, and maximal respiration; of these, only the reduction in basal respiration was modestly rescued by SSOE (Fig. 7C). Meanwhile, the marked TGF-β_2_-induced increase in glycolysis over time was potently suppressed by SSOE, while glycolytic capacity and reserve remained stable (Fig. 7D). Together, these results demonstrate that inflammatory hypoxia drives the bioenergetic reprogramming toward a compensatory glycolysis shift underlying the EMT of RPE cells, which is counteracted by local oxygenation.

## DISCUSSION

In this study, we identify cellular hypoxia as an early, sustained, and mechanistically pivotal driver of retinal fibrosis in PVR. Integrating human single-cell transcriptomics with a clinically relevant ocular trauma model, we demonstrate that hypoxia arises rapidly after injury, persists across major disease-associated cell populations, and promotes metabolic and phenotypic reprogramming that underpins EMT and fibrotic progression. Local oxygen delivery using an intraocular supersaturated oxygen emulsion preserved neuroretinal function and potently suppressed fibrocellular membrane formation, demonstrating that hypoxia is not merely a byproduct of tissue injury and fibrosis, but rather a primary, therapeutically actionable pathogenic mechanism. Furthermore, these mechanistic insights likely extend to other blinding intraocular fibrotic diseases associated with inflammatory hypoxia and EMT, including DR and AMD^11,14,52,53^.

Our study establishes a robust translational bridge by pairing a high-fidelity traumatic mouse model with patient-derived primary PVR cell cultures. Unlike commonly used animal models that rely on intravitreal injection of exogenous cells or enzymes, which often fail to form well-characterized fibrocellular membranes on histology examination^23,54,55^ or lack a robust immune-inflammatory response^28^, our OGI model faithfully recapitulates the clinical etiology of traumatic PVR. The resulting pathology closely resembles advanced human grade C PVR, including full-thickness retinal folds and rigid, tractional fibrocellular membranes^35^. Complementing this, primary human PVR cultures derived from surgically excised patient membranes retain spontaneous, clinically representative profibrotic behaviors *in vitro*^56^, providing a highly relevant human system to validate our therapeutic and mechanistic findings.

Our data expand upon recent transcriptomic studies implicating hypoxia-associated signaling in PVR^23^ by providing direct spatial and temporal validation of tissue hypoxia and establishing its functional contribution to fibrotic progression. Whereas prior bulk or low-resolution analyses suggested activation of hypoxia-responsive programs in detached retina and PVR membranes, our integrated single-cell analysis demonstrates that hypoxia signatures are broadly and convergently elevated across multiple disease-associated cellular compartments, including myeloid cells, RPE cells, fibroblasts, Müller glia, and T cells. Notably, the deployment of an *in vivo* molecular hypoxia probe enabled direct capture of hypoxia that emerges early after injury within regions of inflammatory infiltration and retinal disruption.

Our study further advances the therapeutic concept of oxygen modulation in retinal fibrosis through localized intraocular oxygen delivery. We demonstrate that a single intravitreal administration of SSOE safeguards retinal structure and preserves visual function while drastically reducing membrane formation. The inclusion of longitudinal ERG tracking provides functional evidence that restoration of local oxygenation directly benefits neuroretinal signaling, in addition to limiting structural pathology. Taken together, these data firmly establish inflammatory hypoxia as a therapeutically actionable upstream driver of retinal fibrosis.

RPE transdifferentiation via EMT is widely recognized as a central mechanism driving PVR membrane formation^57,58^. EMT is also a conserved mechanism in organ fibrosis and cancer progression^59,60^ and is energetically demanding. Our transcriptomic profiling revealed that human PVR cells exhibit a massive, coordinated upward shift in both EMT and glycolytic pathways. Using human RPE cell cultures, we demonstrate that early-stage EMT is characterized by an initial spike in mitochondrial respiration and rapid intracellular hypoxia. This is followed by severe mitochondrial network fragmentation, a collapse of oxidative phosphorylation, and a classic metabolic shift toward compensatory glycolysis. This metabolic transition represents a critical bioenergetic adaptation to survive localized hypoxic stress^59,60^ and directly parallels observations linking mitochondrial dysfunction to EMT in tumor biology^50^. While experimental conditions may influence the timing of metabolic shifts^51^, the transition toward glycolysis during EMT was consistently observed in our system. Given that pharmacologically boosting mitochondrial pyruvate utilization has been shown to suppress RPE EMT^61^, our findings position inflammatory hypoxia as a crucial upstream trigger that couples mitochondrial disruption to glycolytic reprogramming and the acquisition of profibrotic EMT phenotypes.

More broadly, tissue hypoxia is increasingly recognized as an important driver of fibrotic remodeling across multiple major organ systems, including the lung, liver, kidney, and heart^19,20,62,63^. Hypoxia-associated metabolic reprogramming and mesenchymal transition represent conserved, fundamental pathways underlying progressive fibrosis^60,63–65^. Consequently, our findings suggest that interruption of hypoxia-induced metabolic and phenotypic shifts could serve as a generalizable antifibrotic strategy. While localized delivery methods must be customized for different anatomical sites, the success of local intraocular oxygen supplementation in modulating cellular metabolism and suppressing EMT underscores the vast therapeutic potential of targeting inflammatory hypoxia in chronic fibrotic diseases.

In summary, we establish cellular hypoxia as a critical, upstream driver of retinal fibrosis and provide mechanistic and preclinical evidence that targeted restoration of local tissue oxygenation can prevent or attenuate PVR and related fibrotic eye diseases.

## METHODS

### Mice

Wild-type BALB/c mice (The Charles River Laboratories, Strain #: 028, Wilmington, MA) at 8–10 weeks of age were used for this study. All animal experiments were approved by the Schepens Eye Research Institute Animal Care and Use Committee and adhered to the Association for Research in Vision and Ophthalmology Statement for the Use of Animals in Ophthalmic and Vision Research. Unilateral OGI of the eye was performed in BALB/c mice to induce PVR. Following anesthesia and pupil dilation, a stab incision was made at the peripheral cornea (sparing the limbus) temporally. The corneal incision was extended to approximately 180°, and the lens and anterior vitreous body were gently removed, followed by filling the vitreous cavity with viscoelastic (DuoVisc^®^, Alcon, Fort Worth, TX) to restore the eye shape. The corneal incision was closed with interrupted 11 − 0 nylon sutures (MicroSurgical Technology, Redmond, WA), followed by filling the anterior chamber with viscoelastic again to restore the eye shape and proper intraocular pressure. 5 μl of SSOE, vehicle, or PBS (Corning^®^, Corning, NY) was immediately injected into the viscoelastic-filled eye. A single application of triple antibiotic eye ointment (Bausch & Lomb, Tampa, FL) was applied topically to the injured eye after the procedure, then 4 times daily for 72 hours.

### SSOE formulation

Coruna Medical, Inc (Longmont, CO) provided a formulation of SSOE with 15% PFD and its unoxygenated vehicle containing the following ingredients: distilled water, hydrogenated phosphatidylcholine (American Lecithin Company, Oxford, CT), polawax NF (Croda Inc, Edison, NJ), and PFD (Mel-Co^®^, Coachella Valley, CA).

### Electroretinography (ERG)

Following overnight dark adaptation, mice were prepared for ERG recording under dim red light. After anesthesia and pupil dilation, one small amount of sterile GenTeal lubricating gel (Alcon) was applied to the cornea to prevent dehydration and to allow electrical contact with the recording electrode (gold wire loop). A 25-gauge platinum needle, inserted subcutaneously in the forehead, served as the reference electrode, while a needle inserted subcutaneously near the tail served as the ground electrode. A series of flash intensities was produced by Espion Ganzfeld (Diagnosys, Lowell, MA) to test both scotopic (dark-adapted) and photopic (light-adapted) responses. The major ERG components (a-wave and b-wave) were measured using the Espion software (Diagnosys). The a-wave amplitude was measured from the baseline to the trough of the a-wave, and the b-wave amplitude was measured from the trough of the a-wave to the peak of the b-wave.

### Tissue and cellular hypoxia detection

Pimonidazole hydrochloride-based kit (Hypoxyprobe-Red549 Kit, Hypoxyprobe, Inc., Burlington, MA) was used. For *in vivo* detection, pimonidazole was injected intraperitoneally at a dose of 1.2 mg/20 g of mouse body weight 90 min before euthanization and eyeball collection, followed by immunofluorescence staining. For *in vitro* detection, cultured cells were incubated with pimonidazole (10 mg/mL) for 1 hour before fluorescent immunocytochemistry staining.

### Tissue immunofluorescence staining

Paraformaldehyde-fixed, paraffin-embedded mouse eyeball sections were deparaffinized with xylene (Millipore Sigma, St Louis, MO) for 30 min, and rehydrated through a graded ethanol series (100%, 96%, 80%, and 70%, Millipore Sigma) and distilled water. Then the samples were permeabilized by 0.5% Triton X-100 (Millipore Sigma) for 30 min and incubated with trypsin antigen retrieval solution (Abcam, Waltham, MA) for 20 min. After washing three times with PBS and blocking with 1% bovine serum albumin (BSA, Millipore Sigma) for 60 min, and the samples were incubated overnight at 4°C with the primary antibodies specifically against α-SMA (ab124964, 1:100, Abcam), collagen I (PA5-95137 , 1:100, ThermoFisher, Waltham, MA), vimentin (PA5-27231, 1:100, ThermoFisher), or with the primary RED 549 dye-MAb1 against pimonidazole (Hypoxyprobe-Red549 Kit, 1:100) or Alexa Fluor 594-anti-mouse F4/80 antibody (123140, 1:100, BioLegend, San Diego, CA). Samples incubated with unconjugated primary antibodies were then incubated with the Alexa Fluor 488-goat anti-rabbit IgG (H + L) secondary antibody (A11008, 1:200, ThermoFisher), followed by nuclear counterstaining with DAPI (ThermoFisher). Samples were then washed with PBS and mounted on a slide using the VECTASHIELD mounting medium (Vector Laboratories, Newark, CA). Images were acquired using a Leica TCS SP8 confocal microscope (Leica Microsystems Inc., Buffalo Grove, IL). The fluorescence-stained areas or cells were quantified using ImageJ (National Institutes of Health, Bethesda, MD).

### TUNEL staining

Eyeball sections were deparaffinized and permeabilized as described above, and then blocked with 1% BSA in PBS containing 0.05% Tween-20 (PBST) for 30 min at room temperature. Apoptotic nuclei were detected using the In Situ Cell Death Detection Kit, TMR red (Millipore Sigma) in accordance with the manufacturer’s protocol. Briefly, sections were incubated with 50 μL/slide of terminal deoxynucleotidyl transferase (TdT)-containing TUNEL reaction mixture at 37°C for 60 min in a humidified chamber protected from light. Following the labeling reaction, slides were washed thoroughly in PBS to remove unincorporated fluorophore. Nuclei were counterstained with DAPI in the mounting medium, and sections were cover-slipped. Images were acquired using a Leica TCS SP8 confocal microscope.

### Fluorescent immunocytochemistry staining

Cells grown on coverslips in 24-well plates were washed with PBS, fixed with 4% paraformaldehyde (Millipore Sigma) for 15 min, and then permeabilized with 1% Triton X-100 for 5 min. Cells were blocked with 1% BSA in 0.5% PBST for 1 hour at room temperature, and incubated overnight at 4°C with the following primary antibodies: anti-α-SMA (1:200), anti-collagen I (1:200), anti-vimentin (1:200), anti-fibronectin (15613-1-AP, 1:200, Proteintech, Rosemont, IL), anti-E-cadherin (AF748, 1:200, R&D Systems, Minneapolis, MN), Alexa Fluor 594 anti-ZO1 (33914, 1:200, ThermoFisher), and RED 549 dye-MAb1 against pimonidazole (1:100). Cells incubated with unconjugated primary antibodies were further incubated with Alexa Fluor 488-goat anti-rabbit IgG (H + L) secondary antibody (1:200) or FITC-conjugated donkey-anti-goat antibody (705-095-147, 1:200, Jackson ImmunoResearch Labs, West Grove, PA) for 1 hour at room temperature, followed by nuclear counterstaining with DAPI. Coverslips were mounted using mounting medium and examined with a Leica SP8 confocal microscope. The fluorescence intensity was quantified using ImageJ cell counter plugin.

### Retinal wholemount immunofluorescence staining

Mouse eyeballs were fixed with 4% paraformaldehyde at room temperature for 1 hour and carefully trimmed to remove the optic nerve and excess extraocular tissue. Eyecups were prepared by removing the anterior segment, and the neural retina was carefully dissected. After washing with PBS, retinas were flattened with four incisions from the periphery towards the optic nerve head, and then stained with a TUNEL kit (25879, Cell Signaling Technology, Danvers, MA) according to the manufacturer’s instructions. Briefly, retinas were washed with PBS, permeabilized with 0.5% PBST, and washed twice with PBS. For positive control, retinas were incubated with DNase (Qiagen) for 10 min. Samples were incubated in TUNEL equilibration buffer for 5 min, followed by TUNEL reaction mixture for 60 min at 37°C. After PBS washes, retinas were blocked with 10% fetal bovine serum (FBS, Gibco, ThermoFisher) for 2 hours at room temperature and incubated with a primary antibody against Brn3a (MAB1585, Millipore Sigma) overnight at 4°C. Samples were washed and incubated with the secondary antibody Alexa Fluor 594 goat anti-mouse (A-110005, 1:200, ThermoFisher) at 4°C overnight. Samples were then washed with PBS and mounted on a slide using the mounting medium. Images were acquired using a Leica TCS SP8 confocal microscope, and analyzed using ImageJ.

### Hematoxylin and eosin staining and Masson’s Trichrome staining

For histomorphology assessment, eyeballs were fixed in 10% formalin, dehydrated through graded ethanol, embedded in methacrylate, cross-sectioned, and stained with hematoxylin and eosin (H&E). Masson’s Trichrome staining was performed using a commercial staining kit (ab150686, Abcam) in accordance with the manufacturer’s instructions. Briefly, paraformaldehyde-fixed, paraffin-embedded mouse eyeball sections were deparaffinized and rehydrated. Sections were incubated in Bouin’s solution, followed by nuclear staining with Weigert’s iron hematoxylin. Cytoplasmic staining and collagen differentiation were performed using Biebrich scarlet-acid fuchsin, phosphomolybdic/phosphotungstic acid, and aniline blue. Slides were subsequently differentiated with acetic acid, dehydrated through ethanol, cleared in xylene, and mounted with synthetic resin. Whole-slide brightfield imaging of H&E and Masson’s staining was performed using a NanoZoomer digital slide scanner (Hamamatsu Photonics, Hamamatsu, Japan).

### Primary human cell culture

Primary human retinal pigmented epithelial cells (HRPE) (Catalog #6540, ScienCell Research Laboratories, Carlsbad, CA) were grown in a poly-L-Lysine (ScienCell Research Laboratories) coated surface in Epithelial Cell Medium (EPiCM, containing 2% FBS, 1% Epithelial Cell Growth Supplement, and 1% penicillin/streptomycin) (ScienCell Research Laboratories). Primary human trabecular meshwork cells (HTMC) (Catalog #6590; ScienCell Research Laboratories) were grown in a poly-L-Lysine-coated surface in Trabecular Meshwork Cell Medium (TMCM, containing 2% FBS, 1% Trabecular Meshwork Cell Growth Supplement, and 1% penicillin/streptomycin solution) (ScienCell Research Laboratories). Primary human retinal microvascular endothelial cells (HRMEC) (Catalog # ACBRI-181, Cell Systems, Inc., Kirkland, WA) were cultured in Complete Classic Medium With Serum and CultureBoost (4Z0–500, Cell Systems, Inc.). Cell media was changed every 3 days, and cells were passaged at 90% confluence. All experiments were performed using p5-p8 cells.

### In vitro cytotoxicity evaluation

At 90%–100% confluency, primary human cells were treated with 0, 1:100, 1:200, and 1:300 concentrations of SSOE or its vehicle, and images were captured at 24, 48, and 72 hours using a Leica S40 microscope (Leica Microsystems Inc.). For MTT assay, cells were seeded in a 96-well plate at a density of 10,000 cells per well and cultured for 2 days, followed by treatment with 0, 1:100, 1:200, and 1:300 concentrations of SSOE or its vehicle. MTT assay was performed using a commercial kit (Catalog # CT01, Millipore Sigma) according to the manufacturer’s instructions at 24, 48, and 72 hours. The absorbance was measured at 560nm using a Synergy 2 Microplate Reader (BioTek Instruments, Winooski, VT). The measured absorbance value was used to calculate % cell viability relative to the control (no SSOE or vehicle exposure).

### In vitro human ARPE-19 cell-based EMT model

The human ARPE-19 cell line (American Type Culture Collection [ATCC], passage 19, Manassas, VA) were maintained in Dulbecco’s modified Eagle’s medium nutrient mixture F12 (DMEM-F12) (ATCC) containing 10% FBS and 1% penicillin/streptomycin (Gibco). Cell media was changed every 3 days, and cells were passaged at 90% confluence. All experiments were performed using p5-p16 cells. To induce EMT, cells at 90%–100% confluency were treated with human recombinant TGF-β_2_ (5 ng/mL, PeproTech, ThermoFisher) in the presence of SSOE or vehicle (1:200) for up to 48 hours. Images were captured at 24 and 48 hours using a Leica S40 microscope for morphological evaluation. Computer-based analysis of individual cell morphology was performed with ImageJ using the measurement function, as reported earlier^66^. Briefly, the cell was outlined using a polygonal selection, and the cellular area and perimeter were measured, along with the ellipse. The circularity was calculated using the following formula: 4π (area of the cell)/cell perimeter^2^. The aspect ratio was determined by dividing the minor axis by the major axis of the cells.

### Cell migration assay

When the human APRE-19 cells reached 90%–100% confluency, a scratch wound was created using a sterile 1 mL pipette tip, followed by washing with medium to remove detached cells. Cells were treated with human recombinant TGF-β_2_ in the presence of SSOE or vehicle (1:200) for 24 hours. Images were captured using a Leica S40 microscope. Wound closure was quantified with ImageJ, and the percentage of wound healing was calculated relative to the 0-hour time point.

### Collagen gel contraction assay on ARPE-19 cells

The 96-well plates were preincubated overnight with 5 mg/mL BSA at 37°C, followed by removal of the BSA. The ARPE-19 cells were resuspended at a cell density of 1 × 10^6^ cells/mL in collagen gel solution, which consists of 0.5 mL collagen-I solution (3 mg/mL, Corning), 0.2 mL PBS, 0.23 mL DMEM-F12 (containing TGF-β_2_, TGF-β_2_ + SSOE, or TGF-β_2_ + vehicle), and 0.17 mL NaOH (0.1 M, ThermoFisher). After gentle mixing, 100 μL of the cell–collagen mixture was transferred to the precoated 96-well plate and incubated at 37°C for 1 hour to facilitate gelification. After the mixture is gelled, an additional 100 μL of the respective media with different treatments was added to the corresponding gel groups. Images were captured using a Leica S9i microscope (Leica Microsystems Inc.) at 24, 48, and 72 hours. The percentage of gel contraction was analyzed relative to the 0-hour time point using ImageJ.

### Primary human PVR cell culture and gel contraction assay

The primary cells were isolated from PVR patient membranes, and the cell culture was previously established in our laboratories^26^ in the culture medium that contains MCDB 131 Medium (Gibco) supplemented with 10% FBS, 1% GlutaMAX (Gibco), 1% penicillin/streptomycin, and EGM^®^-2 Endothelial SingleQuots^®^ Kit (Lonza). Gel contraction assay was performed using the CytoSelect^™^ 48-Well Cell Contraction Assay Kit (Cell Biolabs Inc., San Diego, CA). In brief, cell contraction matrix was prepared as described in the kit and mixed with cells (2 × 10^6^/mL) suspended in culturing medium with SSOE or its vehicle. 250 μL of the mixture was added to each well of the 48-well plate. The culture plate was incubated for 1 hour at 37°C to allow the gel to solidify. Thereafter, an additional 500 μL of the respective media with different treatments was added to the corresponding gel groups. Images were captured using a Leica S9i microscope (Leica Microsystems Inc.) at 24 hours. The percentage of gel contraction was analyzed relative to the 0-hour time point using ImageJ.

### Cell mitochondrial morphology imaging and automated 3D analysis

MitoTracker Orange CMTMRos (ThermoFisher) was reconstituted to a 1 mM stock in DMSO (Millipore Sigma) and diluted to 250 nM in serum-free DMEM-F12 medium and incubated for 30 min at 37°C. ARPE-19 cells were fixed with 4% paraformaldehyde for 15 min and then washed and permeabilized with 1% Triton X-100 in PBS (PBST) for 5 min. Cells were then incubated with DAPI for 10 min to counterstain nuclei. Coverslips were mounted using the mounting medium and examined with a Leica SP8 confocal microscope. The 3D analysis of mitochondria morphology and network characteristics was performed using ImageJ (1.52q) as reported earlier^51,67^. Briefly, the image stacks were deconvoluted, pre-processed using several Java-based functions, including background subtraction, sigma filtering, local contrast enhancement, and gamma correction to remove noise from the biostructure signal. The pre-processed images were then reviewed using a proofing-sheet to optimize the parameters of local adaptive threshold algorithm, including block size and C-value. Using batch-processing functionality, all images were thresholded and binarized to isolate the mitochondrial network biostructure signal, using optimized configuration parameters. Once the mitochondrial network biostructures were isolated, thresholded images were post-processed using Despeckle and Remove Outliers to remove residual noise, and then the 3D Fill Holes command was applied to make a 3D stack. The processed images were analyzed using the 3D Object Counter to count mitochondrial objects. The Particle Analyzer 3D command, part of the MorphoLibJ package, was used to generate mitochondrial parameters, including sphericity, volume, and surface area, and the Skeletonized 3D Network Analysis command was used to generate mitochondrial network connectivity parameters, including branch number, length, and diameter.

### Seahorse extracellular flux analysis

Real-time oxygen consumption rate (OCR) and extracellular acidification rate (ECAR) were measured using the Seahorse XFe96 Extracellular Flux Analyzer (Agilent Technologies, Santa Clara, CA). ARPE-19 cells were seeded on Seahorse XF96/XF Pro cell culture microplates at a density of 2 × 10^4^ cells per well and cultured for 24 hours in DMEM-F12 medium, followed by treatment with human recombinant TGF-β_2_ in the presence of SSOE or vehicle for 48 hours. Subsequently, for the Mito Stress Test, cells were incubated for 1 hour in a CO_2_-free humidified incubator at 37°C with assay medium composed of Seahorse XF Base Medium (pH 7.4, Agilent Technologies) without phenol red, containing 1 mM pyruvate (Gibco), 2 mM L-glutamine (Gibco), and 25 mM glucose (Millipore Sigma). The following reagents from the Seahorse XF Cell Mito Stress Test kit (Agilent Technologies) were sequentially injected at 16, 36, and 56 min with final concentrations of 1 μM oligomycin, 1.5 μM carbonyl cyanide-p-trifluoromethoxy phenylhydrazone (FCCP), and 1 μM rotenone/antimycin A, respectively. For the Glycolytic Stress Test, cells were incubated in a similar environment, with assay medium composed of Seahorse XF Base Medium containing 2 mM L-Glutamine alone. The following reagents from the Seahorse XF Glycolysis Stress Test kit (Agilent Technologies) were sequentially injected at the same time points as the Mito Stress Test with final concentrations of 10 mM glucose, 2 μM oligomycin, and 50 mM 2-deoxyglucose (2-DG). All OCR and ECAR values were normalized to the individual protein concentrations in each well, measured by a BCA assay kit (ThermoFisher).

### Bioinformatic analyses of human scRNA-seq datasets

We analyzed three previously published human scRNA-seq datasets spanning healthy retinal tissues and PVR membranes. PVR data were obtained from the Broad Single Cell Portal (SCP2582)^26^. Healthy RPE/choroid reference data were obtained from Voigt et al. (GEO: GSE135922; ArrayExpress: E-GEOD-135922)^25^, and healthy neuroretina reference data were obtained from the adult human retina single-cell atlas by Lukowski et al. (ArrayExpress: E-MTAB-7316)^27^. Analyses were performed in R (4.5.2) using the following packages: Seurat/SeuratObject (core single-cell processing and scoring), Matrix (sparse matrices), data.table (fast I/O), dplyr (1.1.4), ggplot2 (4.0.2), cowplot (1.2.0), scales (1.4), ggrepel (0.9.6), and msigdbr (25.1.1 MSigDB gene set import). Datasets were processed independently using the Seurat framework in R^68^. Cells were filtered using uniform quality-control thresholds (≥ 200 detected genes, ≥ 500 UMIs, ≤ 25% mitochondrial transcripts), followed by log-normalization, selection of 3,000 variable genes, scaling, principal component analysis, and UMAP visualization. The RPE/choroid dataset served as the reference for lineage-appropriate comparisons with PVR membranes, while Müller glia cells were compared against the healthy neuroretina reference. Cell types were annotated using ScType with validation by canonical lineage markers and harmonized into major lineage classes to enable cross-dataset comparisons, and results were visualized through UMAP projections, marker dot plots, and cell composition summaries. Hypoxia pathway activity was quantified using the MSigDB Hallmark gene sets hypoxia signature^69^, generating per-cell module scores and donor-level median scores to avoid pseudoreplication bias^70^. Statistical comparisons between Healthy and PVR samples were performed using Wilcoxon rank-sum tests with Benjamini–Hochberg correction. For global pathway overview analyses, donor-pseudobulk profiles were generated using all cells from each dataset and analyzed across all Hallmark gene sets. Pathway effect sizes were calculated as the median ssGSEA score in PVR minus the median score in the matched healthy reference.

### Statistics

Statistical analysis was performed using GraphPad Prism 10.6.0, with p-values < 0.05 considered statistically significant. A one-way ANOVA or a mixed-effects analysis with Tukey’s post hoc multiple comparisons test was used. Results are presented as mean ± SD.

## Supplementary Material

Supplementary Files

This is a list of supplementary files associated with this preprint. Click to download.

• SupplementaryMaterials.docx

## Data Availability

Values for all data points in graphs are reported in the Source Data file.
